# CDK10 loss primes tumor cells for transcriptional vulnerability and sensitizes to CDK12/13 inhibition

**DOI:** 10.1038/s41419-026-08973-x

**Published:** 2026-06-11

**Authors:** Shafiqul Islam, Yusuke Tarumoto, Mariko Takano, Seiichi Sugino, Gohei Nishibuchi, Akio Mizutani, Hiroko Yamakawa, Daisuke Morishita, Kosuke Yusa

**Affiliations:** 1 Institute for Life and Medical Sciences, Kyoto University, Kyoto, Japan; 2 Graduate School of Medicine, Kyoto University, Kyoto, Japan; 3 Chordia Therapeutics, Inc, Kanagawa, Japan

**Keywords:** Tumour biomarkers, Tumour biomarkers

## Abstract

CDK12 and CDK13, two transcriptional cyclin-dependent kinases (CDKs), are emerging as therapeutic targets in epithelial cancers due to their vital roles in transcriptional elongation and maintaining genomic stability. However, the absence of predictive biomarkers may impede the clinical use of CDK12/13 inhibitors. Here, we identify CDK10 as a consistent and kinase-dependent regulator of sensitivity to CDK12/13 inhibition. A genome-wide CRISPR-Cas9 screen across six breast and ovarian cancer models reveals CDK10 loss as a top-ranked enhancer of response to the selective CDK12/13 inhibitor CTX-439. Functional validation in multiple cancer cell lines and xenograft models shows that CDK10 deficiency boosts apoptotic responses and increases the antitumor effects of CDK12/13 inhibition. Mechanistically, CDK10 loss extends the duration of CTX-439-induced transcriptional suppression and delays RNA polymerase II re-engagement at sensitive gene loci, exemplified by *CAPN7* and *SENP6*. These findings indicate CDK10 as a probable biomarker for patient stratification and as a co-target to boost the efficacy of transcriptional therapies. Our work reveals a previously underappreciated role for CDK10 in transcriptional resilience, emphasizing its potential to guide and enhance CDK12/13-based cancer treatments.

## Introduction

Cyclin-dependent kinases (CDKs) integrate control of cell division and transcription, and their dysregulation is a recurrent feature of human cancers [1–3]. Within the transcriptional arm of this family, different CDKs act sequentially on the RNA polymerase II (Pol II) carboxy-terminal domain (CTD) to drive distinct stages of the transcription cycle. CDK7 phosphorylates Ser5 and Ser7 to promote initiation and establish promoter-proximal pausing, CDK9 phosphorylates Ser2 (S2) to release Pol II into productive elongation, and CDK12/13 maintain S2 phosphorylation across gene bodies to ensure efficient transcription of long, structurally complex genes [4–6]. Sustained S2 phosphorylation by CDK12/13 is therefore critical for the full-length transcription of DNA damage response (DDR) genes including *BRCA1*, *BRCA2*, *ATR*, and *FANCD2*, thereby safeguarding genome stability and cellular survival [7, 8].

Given their role in DDR and genomic stability, CDK12/13 have emerged as attractive therapeutic targets in cancer, particularly in tumors that retain homologous recombination (HR) proficiency [9]. Pharmacological inhibition of CDK12/13 disrupts transcriptional elongation of DDR genes, inducing a “BRCAness” phenotype [10, 11]. This phenotype, characterized by a defect in DNA repair similar to that seen in BRCA-mutated tumors, sensitizes HR-proficient cells to PARP inhibition or replication stress-inducing agents [12–15]. Inducing this phenotype represents a principal mechanism through which CDK12/13 inhibitors achieve therapeutic efficacy [16]. Several selective CDK12/13 inhibitors, such as THZ531, SR-4835, and CTX-439 we developed [17], have demonstrated efficacy in preclinical models by inducing apoptotic cell death through transcriptional catastrophe mainly [10, 11, 17, 18]. However, clinical translation of CDK12/13 inhibitors may face key challenges, particularly in the absence of predictive biomarkers that can stratify responsive patients [16, 19–21]. Unlike PARP inhibitors, whose development was accelerated by *BRCA1/2* mutations as well-defined synthetic lethal markers, equivalent predictive markers for CDK12/13 inhibition remain largely undefined [20, 22]. Although CDK12 mutation status, gene expression signatures, or transcriptional profiles have been proposed as potential indicators of sensitivity [16, 23], these approaches remain limited in clinical robustness and generalizability. Understanding the genetic and molecular factors that influence response to CDK12/13 inhibition is thus crucial for optimizing therapeutic strategies and identifying combination partners to increase efficacy and overcome resistance.

CDK10 is a relatively little-studied member of the CDK family and functions as an active kinase in complex with Cyclin Q (encoded by *CCNQ*, previously known as *FAM58A*) [24–26]. This complex phosphorylates the ETS2 transcription factor, creating a phosphodegron recognized by the E3 ubiquitin ligase FBXW7, which promotes ETS2 ubiquitination and proteasomal degradation, thereby restraining its transcriptional activity [24, 25]. Beyond transcriptional control, CDK10/Cyclin Q also plays a critical role in ciliogenesis, where its loss disrupts primary cilium formation [24, 26]. Consistent with these roles, germline mutations in either CDK10 or Cyclin Q underlie STAR syndrome, in which both ETS2 dysregulation and defective ciliogenesis contribute to developmental abnormalities, aligning this disorder with features of ciliopathies [25, 26]. In cancer biology, CDK10 downregulation has been linked to resistance to endocrine therapy in ER-positive breast cancer, where loss of CDK10 unleashes ETS2-driven transcriptional programs [24, 27]. Reduced CDK10 expression has also been reported in hepatocellular carcinoma, gastric cancer, and gastrointestinal malignancies, where its loss correlates with enhanced proliferation, invasion, and poor prognosis [28–31]. Despite these observations, CDK10’s broader role in transcriptional control and its potential impact on drug response remain largely unexplored.

In this study, we performed a genome-wide CRISPR-Cas9 knockout (KO) screen across the breast and ovarian cancer cell lines using the selective CDK12/13 inhibitor CTX-439 to uncover genetic dependencies that govern drug response. Among the top sensitizers identified was CDK10, whose loss enhanced cellular vulnerability to CDK12/13 inhibition across multiple models. Mechanistic investigations revealed that *CDK10* KO impairs the transcriptional recovery following CTX-439 treatment, resulting in sustained loss of Pol II S2 phosphorylation, which leads to increased apoptosis. Our findings point to CDK10 as a critical modulator of transcriptional resilience and propose its potential utility as a biomarker for predicting or enhancing responses to transcriptionally targeted cancer therapies.

## Results

### Genome-wide CRISPR-KO screens reveal genes influencing CTX-439 sensitivity

**Fig. 1:**
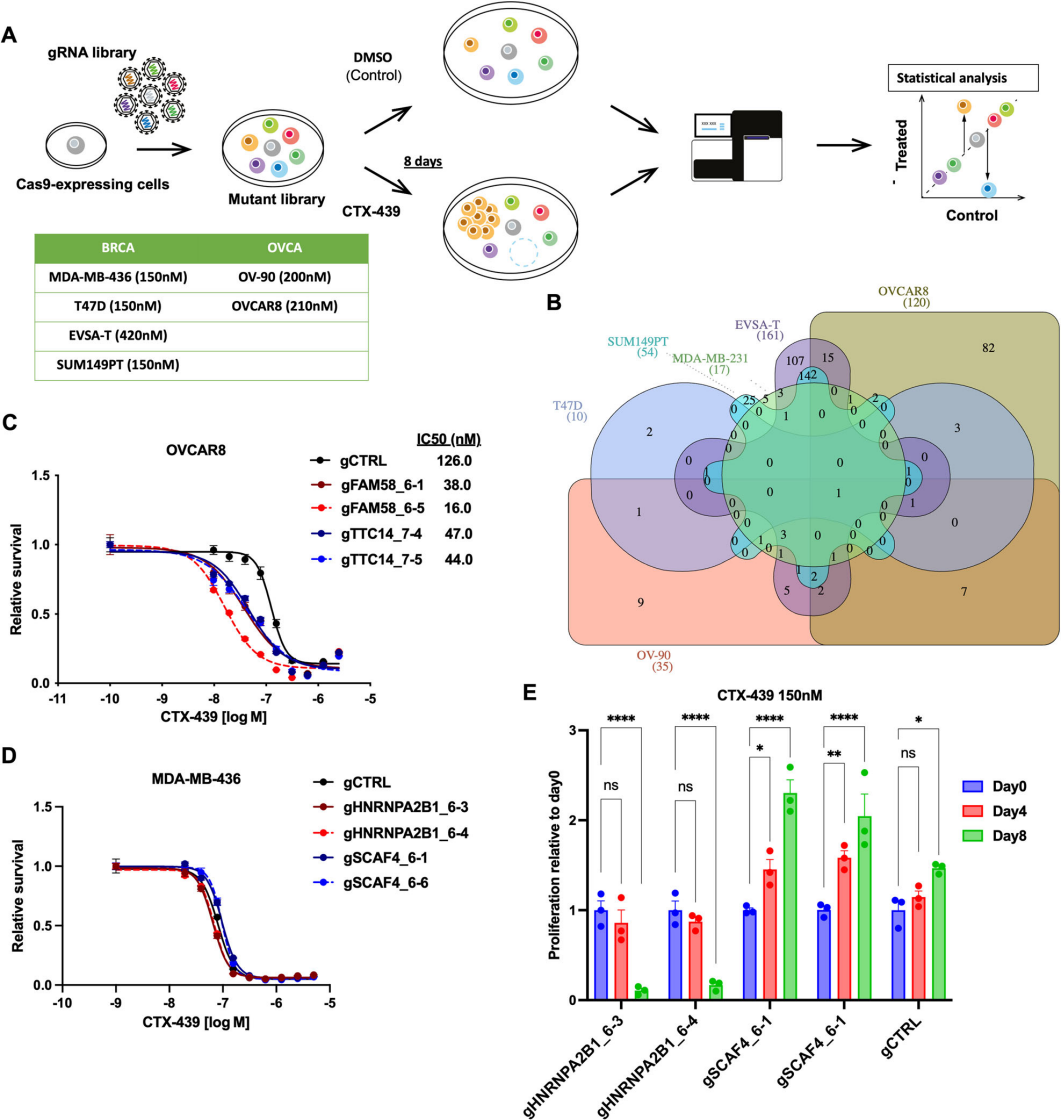
Genome-wide CRISPR-Cas9 screen identifies genetic determinants of CTX-439 sensitivity across cancer models. **A** Schematic of genome-wide CRISPR KO screening performed in six epithelial cancer cell lines (MDA-MB-436, T47D, EVSA-T, SUM149PT, OV-90 and OVCAR8) treated with cytostatic concentrations of CTX-439. **B** Venn diagram showing the overlap of top depleted genes across six cell lines screened. Dose response assay in OVCAR8 cells with KO of *FAM58A* or *TTC14* (**C**) and in MDA-MB-436 cells with KO of *HNRNPA2B1* or *SCAF4* (**D**), treated with CTX-439 for 72 h. **E** Time-course cell proliferation assays performed by cell counting in MDA-MB-436 cells with *HNRNPA2B1* or *SCAF4* KO under CTX-439 treatment. Data are shown as mean ± SD (*n* = 3). Statistical analyses were performed using one-way ANOVA followed by Tukey’s post hoc test for multiple comparisons. *p < 0.05, **p < 0.01, ***p < 0.001, ****p < 0.0001.

To uncover genetic vulnerabilities that modulate the cellular response to CDK12/13 inhibition, we performed a genome-wide CRISPR-Cas9 KO screen across four breast (MDA-MB-436, T47D, EVSA-T and SUM149PT) and two ovarian (OVCAR8 and OV-90) cancer cell lines. The cells were infected with a pooled gRNA library and treated with cytostatic concentrations of the selective CDK12/13 inhibitor CTX-439 for 8 days, followed by next-generation sequencing to quantify gRNA representation under treatment versus vehicle conditions (Fig. 1A).

The analysis identified a robust set of gene depletions or enrichments, which correspond to increased sensitivity or resistance to CTX-439, respectively, upon hit gene disruption. A Venn diagram comparing the depletion hits across the six cell lines revealed 18 genes commonly depleted in at least three models, highlighting potential shared modulators to CDK12/13 inhibition (Fig. 1B and Supplementary Table 1). Among the most recurrent sensitizer genes were *CDK10*, *FAM58A* (encoding Cyclin Q), *CNOT4*, and *TTC14*. CDK10 and FAM58A form a functional kinase complex that governs ETS2 activity and regulates primary ciliogenesis [25, 26]. CNOT4 is a ubiquitin ligase component of the CCR4-NOT complex, which regulates mRNA stability, deadenylation, and decay processes that may become critical under transcriptional stress [32]. TTC14, although less well-studied, has been detected in chromatin-enriched protein networks and may serve as a scaffold for protein interactions during transcription [33].

To ensure the accuracy of the screen, we selected a small number of genes representing depleted (sensitizer) and enriched (resistance-associated) hits, including *FAM58A*, *TTC14*, *HNRNPA2B1*, and *SCAF4*. We generated each KO and performed dose-response assays. *FAM58A* and *TTC14* KOs in OVCAR8 cells significantly reduced the half-maximal inhibitory concentration (IC50) of CTX-439, confirming their role in sensitization (Fig. 1C). In contrast, KOs of *HNRNPA2B1* (sensitizer) and *SCAF4* (resistance) in MDA-MB-436 cells had a minimal impact in short-term viability assays (Fig. 1D). *HNRNPA2B1* encodes a heterogeneous nuclear ribonucleoprotein involved in RNA splicing, stability, and transport, and is known to regulate stress-responsive and DNA repair transcripts [34]. Its loss may not immediately impair viability, but under prolonged CDK12/13 inhibition, which itself induces transcriptional stress, HNRNPA2B1 depletion may exacerbate transcriptional collapse. In fact, the treatment duration in the CRISPR screens was 8 days. Indeed, 8-day proliferation assays revealed that *HNRNPA2B1* KO significantly suppressed cell growth under CTX-439 treatment (Fig. 1E). Conversely, *SCAF4* KO conferred a mild but significant proliferative advantage over time under CTX-439 exposure (Fig. 1E). Together, these results validated some of our screening hits, supporting the robust identification of genes that influence sensitivity to CDK12/13 inhibition by our screens.

### Validation of *CDK10* KO as a CTX-439 sensitizer hit in ovarian cancer (OVCA) cell lines

**Fig. 2:**
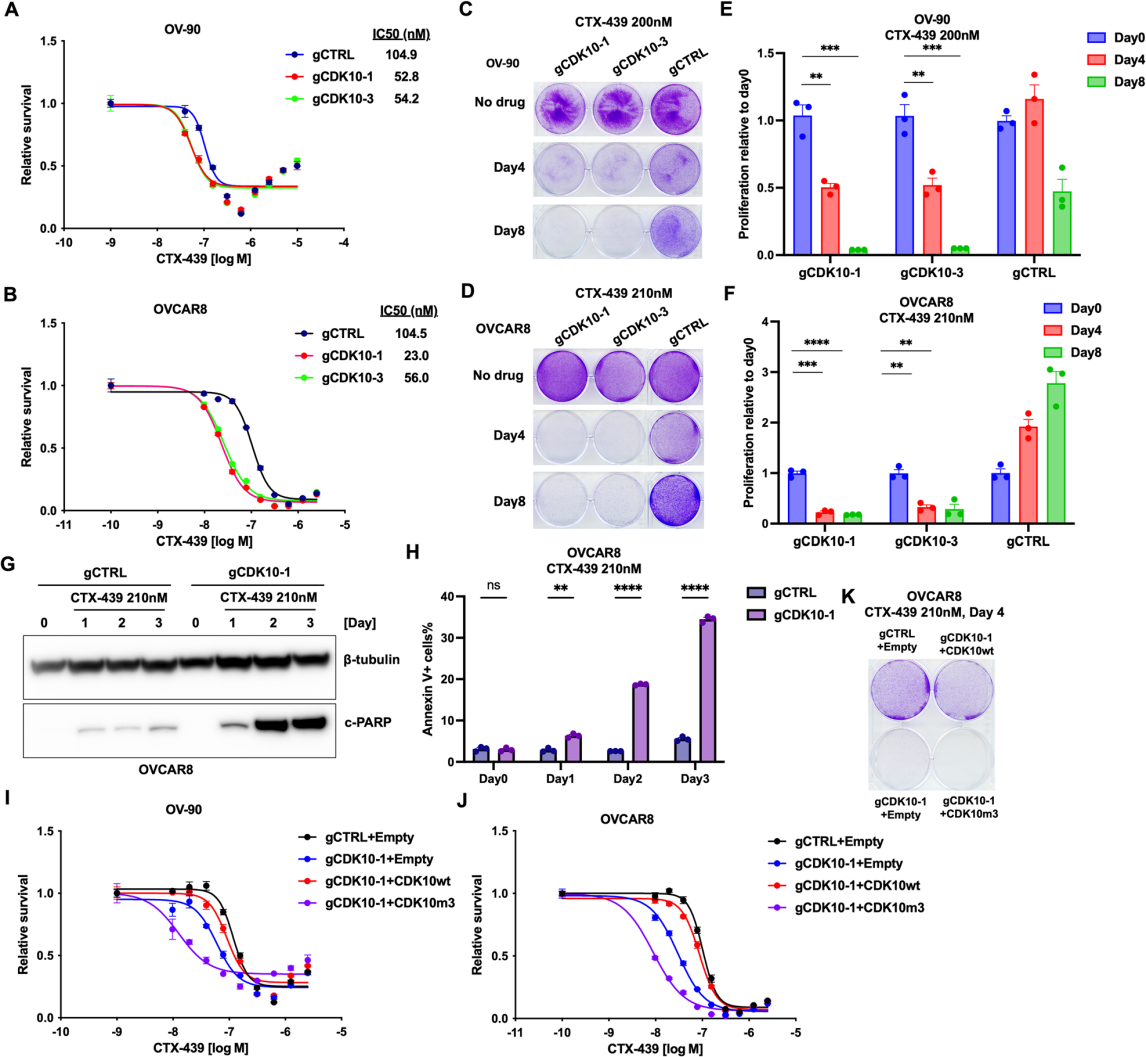
*CDK10* KO potentiates CTX-439 sensitivity in ovarian cancer cells. Dose response assay in *CDK10*-KO and control OV-90 (**A**) and OVCAR8 (**B**) cells treated with CTX-439 for 72 h. Two independent gRNAs against *CDK10* (gCDK10-1 and gCDK10-3) or non-targeting control gRNA (gCTRL) were used. Crystal violet staining of *CDK10-*KO and control OV-90 (**C**) and OVCAR8 (**D**) cells treated with CTX-439 for 4 and 8 days. DMSO controls (No drug) were stained on day 4. Relative proliferation of *CDK10-*KO and control OV-90 (**E**) and OVCAR8 (**F**) cells treated with CTX-439 for the indicated time point. Assays were performed by counting surviving cells. **G** Immunoblot analysis of c-PARP in *CDK10*-KO and control OVCAR8 cells treated with CTX-439 up to 3 days. β-tubulin was used as a loading control. **H** Quantification of apoptosis by Annexin V staining in *CDK10*-KO and control OVCAR8 cells treated with CTX-439 for the indicated days. Dose response assay in OV-90 (**I**) and OVCAR8 (**J**) cells reconstituted with empty vector, CDK10-wt, or kinase-dead CDK10-m3. Cells were treated with CTX-439 for 72 h. **K** Crystal violet staining of OVCAR8 cells treated with CTX-439 for 4 days, after reconstitution with indicated constructs. Data are shown as mean ± SD (*n* = 3). ns not significant; **p < 0.01, ***p < 0.001, ****p < 0.0001 by two-tailed Student’s *t* test.

The fact that FAM58A, the partner cyclin of CDK10, sensitized cells to CDK12/13 inhibition when disrupted strongly indicates CDK10’s involvement. To confirm that CDK10 loss sensitizes cells to CTX-439, we first performed targeted KO experiments in ovarian cancer cell lines using two independent gRNAs (gCDK10-1 and gCDK10-3), both of which fully depleted protein expression (Supplementary Fig. 1A). CDK10 loss did not significantly affect baseline proliferation (Supplementary Fig. 1B). However, in both OV-90 and OVCAR8 models, which were used in our screens, *CDK10* KO significantly enhanced CTX-439 sensitivity, as reflected by a marked leftward shift in dose-response curves and a 50–70% reduction in IC50 values (Fig. 2A, B). We then extended these analyses to ES2 ovarian cancer cells, which were not part of the initial screen, to test the generalizability of CDK10-dependent sensitization. Similar to OV-90 and OVCAR8, *CDK10* KO in ES2 markedly reduced CTX-439 IC50 (Supplementary Fig. 1C). Crystal violet staining confirmed reduced cell viability in *CDK10*-deficient OV-90 and OVCAR8 cells upon drug treatment (Fig. 2C, D), consistent with enhanced cytotoxicity. We further quantified proliferation by cell counting, which showed a marked decrease in cell number in *CDK10*-KO OV-90 and OVCAR8 cells treated with CTX-439 relative to wild-type controls (Fig. 2E, F).

To investigate the apoptotic response, we assessed PARP cleavage by immunoblotting. *CDK10*-deficient cells showed higher cleaved PARP (c-PARP) levels following CTX-439 exposure, indicative of increased apoptosis (Fig. 2G). Flow cytometric analysis further demonstrated a significant increase in Annexin V-positive cells in *CDK10* KO cells compared with controls at 24–72 h post-treatment (Fig. 2H), confirming that CDK10 loss enhances CTX-439-induced apoptosis.

To investigate that the observed sensitivity was explicitly due to CDK10 loss and dependent on the deficiency of its kinase activity, we conducted genetic rescue experiments (Supplementary Fig. 1D). Re-expression of wild-type CDK10 (CDK10-wt) in *CDK10*-KO OV-90, OVCAR8, and ES2 restored the actual response to CTX-439, as reflected by normalized dose-response curves (Fig. 2I, J, Supplementary Fig. 1E). In contrast, the expression of a kinase-dead mutant (CDK10-m3, D181N [35]) failed to rescue the phenotype (Fig. 2I, J and Supplementary Fig. 1E). Interestingly, the kinase-dead CDK10 expression increased sensitivity to CTX-439 more than *CDK10* KO in OV-90 and OVCAR8. Consistently, crystal violet staining showed cell viability recovery only in cells expressing CDK10-wt (Fig. 2K, Supplementary Fig. 1F). Together, these results establish CDK10 as a kinase-dependent modulator of CTX-439 sensitivity in ovarian cancer.

### CDK10 also limits CTX-439 sensitivity in breast cancer (BRCA) cell lines

**Fig. 3:**
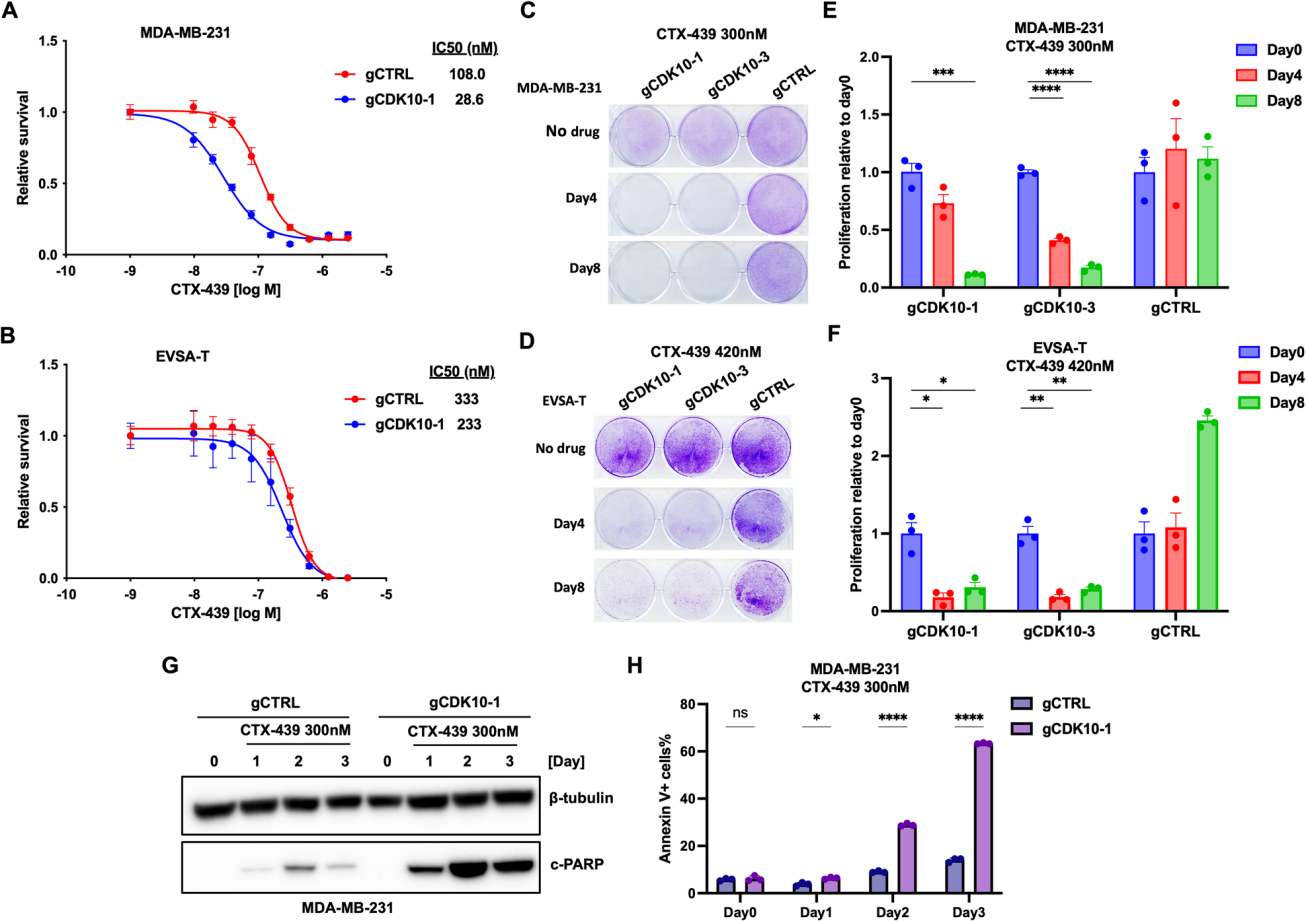
CDK10 loss enhances CTX-439 effectivity in breast cancer models. Dose response assay in MDA-MB-231 (**A**) and EVSA-T (**B**) cells treated with CTX-439 for 72 h following *CDK10* KO (gCDK10-1). Crystal violet staining of *CDK10*-KO MDA-MB-231 (**C**) and EVSA-T (**D**) cells treated with CTX-439 for 4 and 8 days. DMSO controls (No drug) were stained on day 4. Relative proliferation in MDA-MB-231 (**E**) and EVSA-T (**F**) cells treated with CTX-439 for the indicated duration following *CDK10* KO. **G** Immunoblot analysis of c-PARP in *CDK10*-KO and control MDA-MB-231 cells treated with CTX-439 up to 3 days. β-tubulin was used as a loading control. **H** Apoptotic percentage determined by Annexin V flow cytometry in *CDK10*-KO and control MDA-MB-231 cells following CTX-439 treatment at the indicated time points. Data are shown as mean ± SD (*n* = 3). Statistical significance was determined by two-tailed Student’s *t* test. ns not significant; *p < 0.05; **p < 0.01, ***p < 0.001; ****p < 0.0001.

To validate CDK10 loss as a sensitizer to CDK12/13 inhibition in breast cancer, we performed targeted KO experiments in two representative BRCA models (Supplementary Fig. 2A). *CDK10* KO did not alter baseline cell proliferation in MDA-MB-231 (Supplementary Fig. 2B). In both EVSA-T and MDA-MB-231 cells, *CDK10* KO using gCDK10-1 significantly increased sensitivity to CTX-439, as demonstrated by reduced viability in dose response assays (Fig. 3A, B). These findings were further supported by crystal violet staining, which revealed decreased cell survival in the *CDK10*-deficient BRCA cells following CTX-439 treatment (Fig. 3C, D). Cell count assays confirmed that *CDK10*-KO MDA-MB-231 and EVSA-T cells exhibited impaired proliferation under CTX-439 exposure compared to non-targeting controls (Fig. 3E, F). To assess apoptotic responses, we examined PARP cleavage and Annexin V positivity. *CDK10*-deficient MDA-MB-231 cells exhibited increased c-PARP levels following CTX-439 treatment (Fig. 3G), accompanied by a significant increase in Annexin V-positive cells compared to controls (Fig. 3H), indicating enhanced apoptosis.

To confirm the specificity of this phenotype, we again performed rescue experiments in MDA-MB-231 cells (Supplementary Fig. 2C). Re-expression of CDK10-wt in *CDK10*-KO cells restored the normal CTX-439 response in dose response assays and crystal violet staining (Supplementary Fig. 2D, E). In contrast, the expression of the kinase-dead mutant (CDK10-m3) failed to rescue the sensitivity, reinforcing the requirement of CDK10’s kinase activity to mitigate the enhanced sensitivity to CTX-439. These results establish that, in addition to ovarian cancers, CDK10 suppresses CTX-439 sensitivity in breast cancer models.

### *CDK10* KO enhances sensitivity to alternative CDK12/13 inhibitors but not to cisplatin

**Fig. 4:**
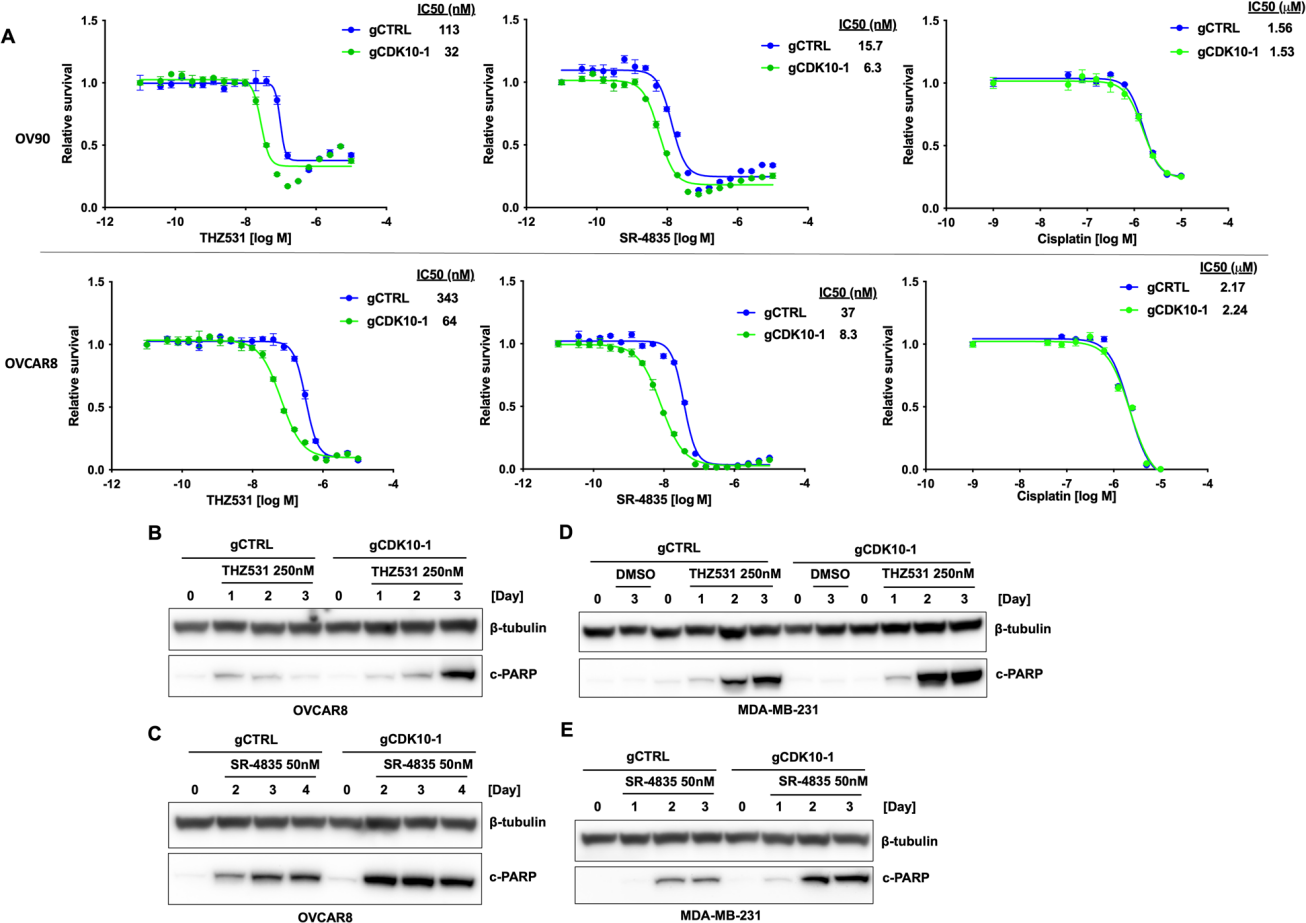
CDK10 selectively modulates response to CDK12/13 inhibitors, but not to DNA-damaging agent, cisplatin. **A** Dose response assay in *CDK10*-KO and control OV-90 and OVCAR8 cells treated with THZ531, SR-4835, or cisplatin for 72 h. Immunoblot analysis of c-PARP in OVCAR8 (**B**, **C**) and MDA-MB-231 (**D**, **E**) cells treated with THZ531 (**B**, **D**) or SR-4835 (**C**, **E**) for the indicated days. β-tubulin was used as a loading control. Data are shown as mean ± SD (*n* = 3).

To determine whether the CDK10-dependent sensitization can be extended to other CDK12/13 inhibitors, we tested the effect of *CDK10* KO in OV-90 and OVCAR8 cells treated with two mechanistically distinct CDK12/13 inhibitors, THZ531 [10] and SR-4835 [11]. Dose-response assays revealed that *CDK10* KO significantly enhanced sensitivity to THZ531 and SR-4835, as shown by a marked leftward shift of dose-response curves (Fig. 4A). In contrast, *CDK10* loss did not alter sensitivity to cisplatin, a DNA-damaging agent, indicating that the observed effect is specific to CDK12 pathway inhibition rather than a general enhancement of cytotoxic stress.

Immunoblotting confirmed that increased sensitivity of *CDK10*-deficient cells to the alternative CDK12/13 inhibitors was also associated with increased apoptosis. OVCAR8 cells lacking CDK10 showed higher c-PARP levels following treatment with either THZ531 or SR-4835 compared to controls (Fig. 4B, C). Similar results were observed in MDA-MB-231 breast cancer cells (Fig. 4D, E).

To assess the generalizability and to extend validation of CDK10’s impact on CDK12/13 inhibitor response, we conducted cell viability assays across a panel of seven BRCA cell lines (Supplementary Fig. 3). This panel included four models not part of the initial CRISPR screen (MDA-MB-468, MCF7, HCC1954, and HCC1187) as well as three models that had been included in the screen (SUM149PT, MDA-MB-436, and T47D). CDK10 protein levels varied among the panel (Supplementary Fig. 3A). *CDK10* KO reduced the IC50 of CTX-439 in several models, although the magnitude of sensitization varied across cell lines (Supplementary Fig. 3B, C). Notably, CDK10 loss did not significantly alter CTX-439 sensitivity in SUM149PT, despite the screen hit, and HCC1187 cells in a 72-h assay. Their insensitivity is likely due to the requirement of longer incubation time (72 h v.s. 8 days for the screen in SUM149PT) or the lower dependence on weakly expressed CDK10 (HCC1187, Supplementary Fig. 3A). Indeed, we observed significantly increased response in SUM149PT in a 6-day assay (Supplementary Fig. 3D). Taken together, these data indicate that CDK10 loss selectively enhances cellular sensitivity to CDK12/13 inhibition and support the broad applicability of CDK10-dependent sensitization within specific cellular contexts.

### *CDK10* KO prolongs CTX-439-induced transcriptional suppression

**Fig. 5:**
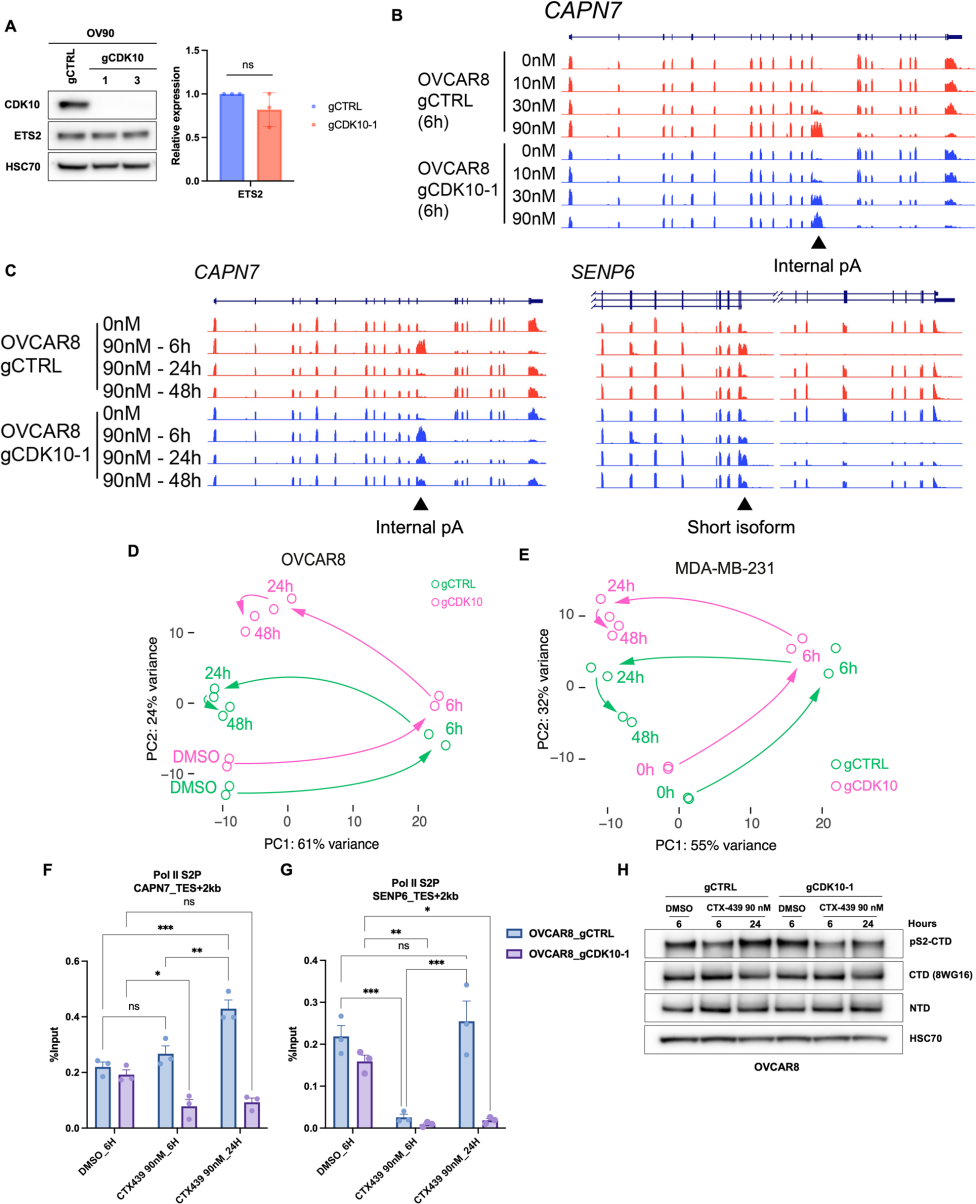
CDK10 supports transcriptional recovery following CDK12/13 inhibition. **A** Immunoblot analysis (Left) of CDK10 and ETS2 in *CDK10*-KO and control OV-90 cells. ETS2 protein levels are quantified (Right). **B** RNA-seq track showing *CAPN7* expression in OVCAR8 cells treated with the indicated concentrations of CTX-439 for 6 h. **C** RNA-seq track showing of *CAPN7* and *SENP6* expression in OVCAR8 cells treated with CTX-439 (90 nM) for the indicated duration. PCA plots of the RNA-seq data from OVCAR8 (**D**) and MDA-MB-231 (**E**) cells treated with CTX-439 (90 nM) for 6, 24, and 48 h. OVCAR8 treated with DMSO for 6 h and MDA-MB-231 at 0 h were included as a control. ChIP-qPCR analysis of Pol II S2P occupancy at transcription end sites (+2 kb from TES) of *CAPN7* (**F**) and *SENP6* (**G**) in OVCAR8 cells treated with DMSO for 6 h or CTX-439 (90 nM) for 6 and 24 h. **H** Immunoblot analysis of RNA Pol II S2P (pS2-CTD), CTD (8WG16) and NTD in *CDK10*-KO and control OVCAR8 cells treated with CTX-439 or DMSO for the indicated duration. HSC70 was used as a loading control. Data are shown as mean ± SD (*n* = 3). Statistical significance was determined using a one-sample *t* test against a hypothetical value of 1 (**A**) or using two-way ANOVA with Tukey’s post hoc test (**F** and **G**). ns not significant; *p < 0.05; **p < 0.01, ***p < 0.001.

Because CDK10/FAM58A has been reported to phosphorylate ETS2 and promote its proteasomal degradation [24], we first assessed ETS2 protein levels following *CDK10* KO. ETS2 expression remained unchanged in OV-90 cells (Fig. 5A), strongly suggesting that CDK10’s modulation of CTX-439 sensitivity occurs independently of ETS2 regulation. We therefore turned to explore alternative mechanisms. Given CDK12’s role in transcriptional elongation and the observation that CDK10 loss did not alter cisplatin sensitivity, we hypothesized that CDK10 more directly influences transcriptional regulation via its kinase activity under CDK12/13-inhibited conditions, and that in the absence of CDK10, CTX-439-induced anti-proliferative effects can be prolonged. To investigate this, we first assessed the subcellular localization of wild-type and its kinase-dead mutant using GFP-tagged constructs. Both forms localized predominantly to the nucleus in OVCAR8 cells (Supplementary Fig. 4A).

We then profiled transcriptional responses in *CDK10* KO and wild-type control cells in OVCAR8 treated with increasing doses of CTX-439 for 6 h by RNA sequencing (RNA-seq). At baseline, *CDK10* KO led to modest transcriptional alterations, with 73 differentially expressed genes, but did not broadly reprogram the transcriptome relative to wild-type controls (Supplementary Fig. 4B). This limited scope of change is consistent with the absence of growth defects in cell count assays (Supplementary Figs. 1B, 2B), indicating that *CDK10* disruption exerts only a minor impact on global transcriptional programs in a CDK12/13-proficient background. Upon CTX-439 treatment, wild-type cells displayed widespread transcriptional alterations, including a dose-dependent reduction in mRNA output alongside the induction of subsets of genes (Supplementary Fig. 4C). At higher concentrations (90 nM), we also observed clear evidence of transcript shortening as illustrated by *CAPN7*, a representative gene that undergoes premature polyadenylation and termination following CDK12/13 inhibition (Fig. 5B). In *CDK10* KO cells, the number of differentially expressed genes was largely comparable to that in wild-type control cells (Supplementary Fig. 4C). However, the transcript truncation was more pronounced in *CDK10* KO cells than in wild-type controls (Fig. 5B). To validate this enhanced truncation, we quantified *CAPN7* exon 12 and intron 12 expression by RT-qPCR (Supplementary Fig. 4D). The intron signal relative to the upstream exon was significantly increased upon 10 nM treatment in *CDK10* KO cells, while remained unaffected in control cells. When comparing genotypes, the intron signal was significantly higher in *CDK10* KO cells than control cells at 10 and 30 nM, although genotype differences were diminished at 90 nM, indicating maximal CDK12/13 inhibition (Supplementary Fig. 4E). To assess transcript shortening genome-wide, we calculated an efficiency evaluation value (EEV, TES depth-to-TSS depth ratio) for each gene using the RNA-seq data obtained from 30 nM treatment (Supplementary Fig. 5A). We observed a lower EEV, particularly in longer genes (>29 kb), in *CDK10* KO cells (Supplementary Fig. 5B). Gene set enrichment analysis on EEV-ranked genes did not identify significant pathway enrichment. These findings suggest that CDK10 is likely to be involved in transcriptional elongation under CDK12/13-inhibited conditions.

The difference in apoptosis induction between wild-type and *CDK10* KO tended to be enhanced more beyond 48 h post-treatment (Figs. 2G and 3G), which may suggest that *CDK10* disruption exerts more impacts on transcription at later stages. To evaluate the temporal dynamics of transcriptional responses, we tracked expression of genes over a 6–48 h window following CTX-439 exposure. Despite the clear transcript truncation of *CAPN7* and *SENP6* at 6 h post-treatment, these genes underwent substantial transcriptional restoration in wild-type cells by 24 h post-treatment and it lasted at least until 48 h post-treatment. In stark contrast, transcript levels remained durably suppressed in *CDK10* KO cells (Fig. 5C). While transcriptional recovery after CDK12 inhibition remains incompletely characterized in the literature, likely due to the limited treatment duration in most studies, our extended time-course suggests that a subset of transcriptionally repressed genes can re-engage over time, strongly indicating the existence of compensatory mechanisms. Our results suggest that CDK10 appears to function, if not fully, in this transcriptional adaptation, consistent with the fact that kinase-dead CDK10 mutant further increased sensitivity in selected cell lines.

To capture the global patterns of transcriptional dynamics, we performed principal component analysis (PCA) of the RNA-seq profiles in OVCAR8 and MDA-MB-231 cells across multiple time points. Under basal conditions (DMSO for 6 h in OVCAR8 and 0 h in MDA-MB-231), PCA revealed minimal global transcriptomic differences between *CDK10* KO and control cells, again indicating that *CDK10* deletion alone does not broadly alter the transcriptome (Fig. 5D, E). Upon treatment with CTX-439, both models exhibited a substantial shift in the global transcriptome state 6 h post-treatment. While control cells began diverging from this suppressed transcriptional signature by 24–48 h, *CDK10* KO cells showed limited advancement along PC1 toward the basal trajectory and instead diverged along PC2, indicating with delayed or impaired transcriptional reprogramming in the absence of CDK10 (Fig. 5D, E). To identify genes durably suppressed in the absence of CDK10, we performed hierarchical clustering of temporal expression trajectories on 1641 genes significantly downregulated at 6 h in both genotypes. Eight clusters were identified, including subsets (Clusters 5 and 8) exhibiting sustained repression especially at 24 h post-treatment in *CDK10* KO cells (Supplementary Fig. 5C, D). We did not detect significant enrichment of KEGG and GO gene sets in any of the 8 clusters, but found that genes in Clusters 5 and 8 are significantly shorter than genes in the other clusters (Supplementary Fig. 5E). Together, these results suggest that CDK10 loss impairs transcriptional recovery through a generalized elongation-associated mechanism rather than pathway-specific regulation.

Given the restoration of transcriptional truncation and global suppression in wild-type cells, it seems that the activity of RNA Pol II is once disrupted by CDK12/13 blockade but can be, at least in part, restored during prolonged treatment. Our results suggest that CDK10 is involved in this restoration. As Pol II activity is strongly correlated with phosphorylation status of its CTD, we performed ChIP-qPCR analysis for S2-phosphorylated Pol II (Pol II S2P) at the transcription end sites (TES + 2 kb) of *CAPN7* and the long isoform of *SENP6*. *CDK10* disruption did not give a significant impact on the Pol II S2P occupancy at these loci. In control cells, Pol II S2P occupancy was either unchanged (*CAPN7*) or reduced (*SENP6*) at 6 h post-treatment. Their levels significantly increased during the next 18 h, resulting in an even higher level (*CAPN7*) or full recovery (*SENP6*), potentially reflected by the restoration of transcriptional truncation. In sharp contrast, *CDK10* KO cells exhibited reduced Pol II S2P occupancy at both loci 6 h post-treatment, and the reduction persistently lasted until at least 24 h post-treatment (Fig. 5F, G). This supports our model in which CDK10 contributes to transcriptional reactivation at repressed genes in CDK12/13-inhibited conditions. Consistent with these locus-specific findings, immunoblot analysis showed that the Pol II S2P level was reduced in both genotypes at 6 h following 90 nM CTX-439 treatment, compared to DMSO controls (Fig. 5H). By 24 h, S2P levels recovered in control cells but remained suppressed in *CDK10* KO cells (Fig. 5H). Together, these results suggest that CDK10 plays a pivotal role in transcriptional resetting following CDK12/13 inhibition, potentially by modulating Pol II phosphorylation directly or indirectly.

### *CDK10* KO enhances the anti-tumor efficacy of CTX-439 in vivo

**Fig. 6:**
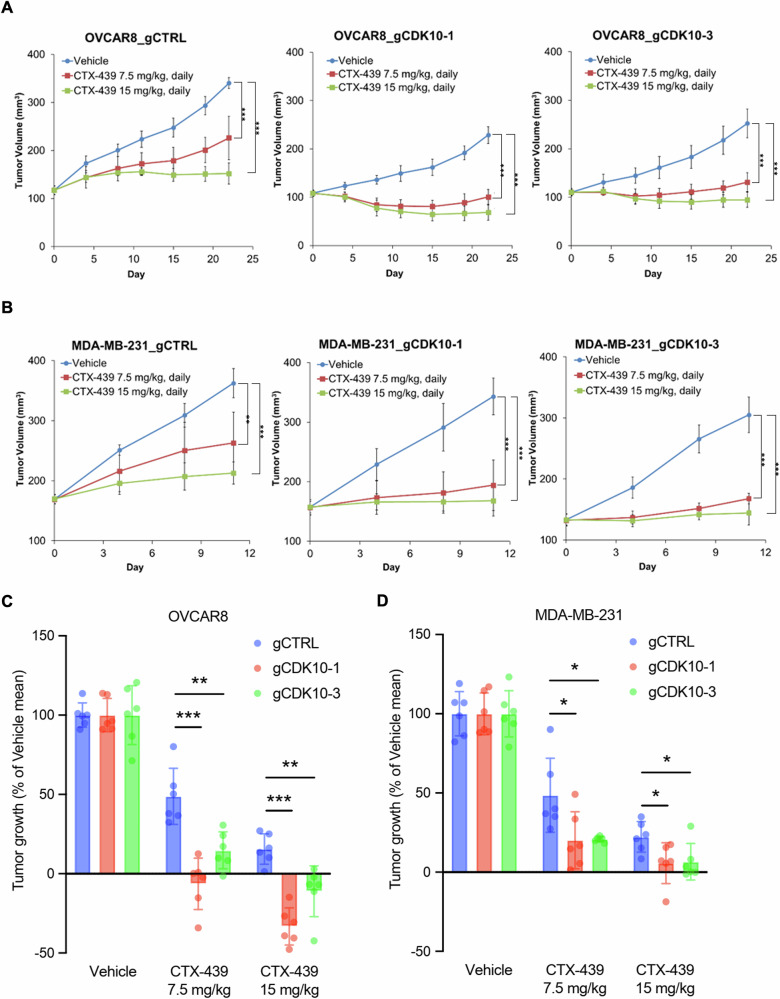
*CDK10* KO enhances in vivo efficacy of CTX-439. Tumor growth curves of OVCAR8 (**A**) and MDA-MB-231 (**B**) xenografts bearing *CDK10* KO (gCDK10-1 or gCDK10-3) or non-targeting control (gCTRL) treated with vehicle or CTX-439 (7.5 or 15 mg/kg, oral gavage, once daily). Tumor volume was measured every 3–4 days. Data are shown as mean ± SD (*n* = 6 mice per group). Treatment groups are compared with the vehicle group at the terminal time point by one-sided Welch’s *t* tests with Holm correction. **C**, **D** Genotype comparisons of tumor growth. Bars show mean ± SD of tumor growth (% of Vehicle) for gCTRL, gCDK10-1, and gCDK10-3 at Vehicle, 7.5 mg/kg, and 15 mg/kg. *CDK10*-KO tumors were compared with the control tumor by one-sided Welch’s *t* tests with Holm correction. *p < 0.05; **p < 0.01; ***p < 0.001.

To determine whether CDK10 loss could potentiate the therapeutic efficacy of CDK12/13 inhibition in vivo, we established xenograft models using OVCAR8 and MDA-MB-231 cells with or without *CDK10* KO. Mice bearing these tumors were treated with CTX-439, and tumor growth was monitored over time. In both models, CTX-439 significantly suppressed tumor growth, compared to vehicle controls (Fig. 6A, B). Importantly, *CDK10* KO tumors exhibited greater growth inhibition than control tumors at both dose levels, and this effect was reproducible across independent gRNAs. Direct genotype comparisons at the terminal time point further confirmed enhanced CTX-439 responsiveness in *CDK10*-deficient tumors (Fig. 6C, D). Together, these in vivo data support our in vitro findings, demonstrating that *CDK10* deficiency augments the anti-tumor efficacy of CDK12/13 inhibition.

## Discussion

Using a genome-wide CRISPR-Cas9 screen across six breast and ovarian cancer models, we uncovered a network of genes whose loss alters response to the selective CDK12/13 inhibitor CTX-439. Among the most recurrent sensitizer hits, CDK10 is considered a consistent determinant of therapeutic vulnerability. Functional validation in both ovarian and breast cancer models demonstrated that *CDK10* KO enhances the cytotoxicity of CTX-439 as well as to other agents in this class. Mechanistic analysis revealed that *CDK10*-deficient cells fail to re-engage RNA Pol II elongation following drug-induced transcriptional blockade, leading to persistent suppression of transcriptionally sensitive genes. These results establish CDK10 as a kinase-dependent modulator of transcriptional resilience and highlight its translational potential as both a biomarker and a therapeutic co-target.

Beyond CDK10’s known roles in ETS2 regulation and ciliogenesis [24–26], its contribution in transcriptional regulation has not been well characterized. Our findings reveal that CDK10 supports transcriptional adaptation under pharmacological stress, positioning it as an auxiliary regulator of transcriptional balance. Unlike canonical transcriptional CDKs, CDK10 does not appear to be required for sustaining global transcription under basal conditions, at least in the contexts we examined. Instead, our data indicate that CDK10 appears critical when cells must sustain transcription during elongation stress. The most plausible molecular mechanism is that CDK10 supports Pol II Ser2 phosphorylation, thereby facilitating transcriptional elongation of CDK12/13-dependent genes. Three lines of evidence support this notion. Firstly, our ChIP-qPCR analysis showed sustained reduction of phospho-S2 abundance in *CDK10* KO as opposed to restoration in wild-type cells. Secondly, kinase-dead CDK10 overexpression exerted dominant-negative effects, sensitizing such cells to CDK12/13 blockade even more than *CDK10* KO cells. Finally, a recent report has shown that recombinant CDK10/Cyclin Q complex can directly phosphorylate Pol II CTD, most efficiently at S2, in vitro [36]. In the same report, however, the phosphorylation target analysis using analog-specific mutant CDK10 exhibited a wide range of substrates involved in transcription, translation, and stress responses [36]. It is therefore possible that CDK10 stabilizes elongating Pol II by modulating accessory factors required for elongation fidelity. Taken together, these insights suggest that CDK10 occupies a previously unrecognized niche within the transcriptional kinase network. We propose that CDK10 serves to buffer transcriptional programs against perturbations of CDK12/13 activity, thereby extending the repertoire of CDKs engaged in maintaining transcriptional resilience.

A major challenge in translating targeted therapies is the heterogeneity of patient response, which can often be addressed by biomarker development [37–39]. A clear example is the use of BRCA mutations and homologous recombination deficiency (HRD) scores to guide PARP inhibitor therapy [12, 21]. Our results nominate CDK10 as such a biomarker, its loss sensitized cells to CDK12/13 inhibitors while sparing sensitivity to DNA-damaging agents such as cisplatin. Recent genomic studies show that CDK10 is frequently deleted or downregulated in subsets of tumors [1, 28–31] and such alterations have been associated with increased tumor growth, invasive behavior, and unfavorable clinical outcomes. We interpret these findings as a collateral, or synthetic, vulnerability. While *CDK10* deficiency may contribute to malignant behavior, it also removes a key transcriptional buffer. As a result, such cancer cells become more dependent on CDK12/13 activity and thus more susceptible to CDK12/13 inhibition, enabling the selection of responsive patients. Importantly, our expanded panel of breast cancer models revealed the variable effect of CDK10 loss. This heterogeneity indicates that CDK10-dependent modulation of CDK12/13 inhibition is context-dependent. Such variability is consistent with the broader landscape of transcriptional dependency in cancer, where lineage state, baseline elongation stress, and compensatory kinase activity may shape therapeutic vulnerability. These findings further underscore the necessity of detailed mechanistic studies to understand how CDK10 is involved in transcription.

In addition to its potential usage as a biomarker, CDK10 may serve as a co-target in CDK blockade therapy. CDK10 proficiency provides buffering capacity against transcriptional stress as shown in CDK12/13 inhibition. In addition to S2 phosphorylation, CDK10 can also directly phosphorylate S5 in vitro [36]. Because transcriptional CDKs act in overlapping but non-redundant pathways [1–3, 40, 41], co-targeting CDK10 with not only CDK12/13 but also other transcriptional CDKs such as CDK7 or CDK9 could destabilize transcriptional homeostasis, enhancing the therapeutic efficacy of CDK blockade. Our CRISPR screen also uncovered other putative sensitizers, such as CNOT4, TTC14, etc., with roles in transcriptional elongation, mRNA stability, and protein interaction dynamics [32–34]. While validation varied across cell lines, these hits reinforce the concept that CDK12/13 inhibitor response is shaped by a complex network of transcriptional, splicing, and post-translational regulators, consistent with recent studies [42–44]. These observations broaden the scope of CDK12/13 biology and point to additional candidates for combinatorial strategies.

While our findings establish CDK10 as a determinant of transcriptional resilience under CDK12/13 inhibition, several questions remain. The precise molecular mechanism by which CDK10 promotes Pol II re-engagement is not fully resolved; whether this occurs through direct phosphorylation of Pol II S2 or through modulation of chromatin-associated cofactors requires further study. Furthermore, the transcriptional restoration by CDK10 seems to be operational at selected loci. A substantial number of affected genes undergo restoration without CDK10 as shown in the PCA analysis (Fig. 5D, E). This locus-specific effect is intriguing and warrants further study.

Under the current study, we demonstrate CDK10 involvement in breast and ovarian cancer models; therefore, it will be important to assess whether CDK10 exerts similar effects in other tumor types, especially those with intrinsic transcriptional vulnerabilities. For example, Ewing sarcoma is driven by the EWSR1-FLI1 fusion oncogene, which imposes a dependence on transcriptional CDKs [18]. Prostate cancers frequently harbor *CDK12* loss-of-function mutations that create reliance on compensatory transcriptional pathways [45]. These tumors may already be sensitive to CDK12/13 blockade but CDK10 co-inhibition could further increase the sensitivity. Addressing these questions will clarify the breadth of CDK10’s role in transcriptional regulation and determine how best to integrate it into biomarker-driven or combination-based therapeutic strategies.

In conclusion, our work identifies CDK10 as a novel determinant of transcriptional resilience and a consistent sensitizer to CDK12/13 inhibition in breast and ovarian cancer. By linking CDK10 to impaired transcriptional recovery and Pol II re-engagement, these findings expand its biological function and highlight its translational relevance. Although pharmacological tools to inhibit CDK10 are still at an early stage, their development will be essential to validate CDK10 as a therapeutic target and to enable rational co-targeting strategies. Incorporating CDK10 into biomarker frameworks and exploring rational combination strategies provide a roadmap for improving the efficacy and durability of transcription-targeted therapies in precision oncology.

## Methods

### Chemical compounds

CTX-439 [17] was provided by Chordia Therapeutics Inc. Additional compounds, including THZ531, SR-4835, and cisplatin, were purchased from MedChemExpress or Selleck Chemicals.

### Cell culture

The SUM149PT breast cancer cell line was obtained from BioIVT and cultured in Ham’s F12 (Thermo Fisher) supplemented with 5% fetal bovine serum (FBS), 10 mM HEPES, 1 μg/mL hydrocortisone, and 5 μg/mL insulin. OV-90 ovarian cancer cells were purchased from ATCC and maintained in DMEM/F12 (Thermo Fisher) supplemented with 10% FBS. Additional breast and ovarian cancer cell lines used in this study are listed in Supplementary Table 2 and described previously [46]. HEK293FT cells were cultured in DMEM (Wako, #041-30081) with 10% FBS. All cell lines were confirmed to be mycoplasma-negative.

### Plasmids and cloning

For Cas9 expression, pKLV2-EF1a-Cas9Bsd-W (Addgene #68343) was used to produce lentivirus [47]. gRNAs (sequences listed in Supplementary Table 4) were cloned into the BbsI site of pKLV2-U6gRNA5(BbsI)-PGKpuro2ABFP-W (Adgene #67974). All gRNA-expressing lentiviral vectors are deposited with Addgene (#246512-246522)

cDNA fragments encoding FLAG-tagged CDK10 WT or kinase-dead (KD, D181N) mutant (referred to as m3) were synthesized and cloned into the BspEI-NotI site of pKLV2-EF1a-neoT2AFlagStop-W (Addgene #246510) using NEBuilder assembly, resulting in pKLV2-EF1a-neoT2AFlagCDK10wt-W (Addgene #246511) and pKLV2-EF1a-neoT2AFlagCDK10m3-W (Addgene #246512), respectively.

To examine CDK10 subcellular localization, CDK10 was cloned with a C-terminal GFP tag into the pKLV2-EF1aNeo vector, yielding pKLV2-EF1aNeoCDK10-GFP.

All constructs were sequence-verified by Sanger sequencing.

### Lentiviral production and transduction

Lentiviruses were produced in HEK293FT cells using Lipofectamine LTX with PLUS Reagent (Thermo Fisher, #15338-100). Each transfection included 900 ng of transfer plasmid, 900 ng of psPAX2 (#12260), 200 ng of pMD2.G (#12259), and 2 μL PLUS reagent in 500 μL OPTI-MEM, followed by addition of 6 μL LTX reagent. Transfection complexes were added to 80% confluent 293FT cells in 6-well plates and incubated for 18 h before medium replacement. Viral supernatants were collected 48 h post-transfection, filtered (0.45 μm), and stored at −80 °C.

For transduction, 1 × 10⁶ cells were incubated overnight with viral supernatant in a 6-well plate. Two to three days later, transduced cells were analyzed by flow cytometry using Attune NxT (Thermo Fisher) and selected with puromycin.

### Generation of Cas9-expressing cell lines

Cas9-expressing cell lines were generated by transducing target cells with lentivirus produced from the pKLV2-EF1a-Cas9Bsd-W vector followed by Blasticidin selection. Functional Cas9 activity was confirmed by transducing cells with pKLV2-U6gRNA5(gGFP)-PGKmBFP2AGFP-W (Addgene #67980) or control empty vector (Addgene #67979), followed by analysis of GFP loss in BFP-positive cells via flow cytometry.

### CRISPR-Cas9 genome-wide screening

Genome-wide CRISPR screening was performed using the human v3 gRNA library targeting 18,740 genes (6 guides/gene, 113,526 guides total) [48]. 3 × 10⁷ cells were transduced at 30% efficiency, selected with puromycin (3–5 μg/mL), and expanded. Approximately 10 days post-transduction, 5 × 10⁷ cells were seeded to T525 flasks (the number of flasks is determined for each cell line depending on seeding density). On the following day, cells were treated with a cytostatic dose of CTX-439 for 8 days, 150 nM for MDA-MB-436, T47D and SUM149PT, 200 nM for OV-90, 210 nM for OVCAR8 and 420 nM for EVSA-T. DMSO-treated controls were run in parallel. Genomic DNA was isolated from DMSO, CTX-439, and day 0 conditions. gRNA abundance was determined via custom primer-based Illumina sequencing (19-bp single-end) and analyzed using MAGeCK [49].

### Dose response assay

Cells (1000/well) were seeded in 96-well plates and treated in triplicate the next day with serially diluted CTX-439, THZ531, SR-4835 or Cisplatin (typically 2-fold, starting at 10 μM) in a constant DMSO background. Cell viability was assessed after 72 h using CellTiter-Glo (Promega) per manufacturer’s instructions. Data were analyzed using GraphPad Prism (v10).

### Proliferation assay by cell count

1–2 × 10^6^ cells were seeded in a 6-well plate and treated the following day with CTX-439 at cytostatic concentrations used in the CRISPR screen (shown in Fig. 1A) or at 300 nM for MDA-MB-231. At the indicated time points, cells were harvested by trypsinization, stained with trypan blue, and counted using a TC20 automated cell counter (Bio-Rad).

### Crystal violet staining

1–2 × 10^6^ cells were plated in a 6-well plate and treated with CTX-439 at the same concentrations used for the cell count assay or 300 nM for ES2 for 4 or 8 days. DMSO treatment was performed for 4 days. Cells were washed with PBS, stained with crystal violet (Nakarai) for 10-15 min, rinsed, and air-dried.

### Immunoblotting

1–2 × 10^6^ cells were seeded in each 10-cm dish. The following day, cells were exposed to CTX-439 at the same concentration used in cell count assay, THZ531 (250 nM), or SR-4835 (50 nM) for the indicated duration. Cells were lysed in RIPA buffer (Sigma, R0278) supplemented with protease-phosphatase inhibitors (Thermo Fisher, 78440). Proteins were resolved using NuPAGE gels (3–8% Tris-Acetate, or 4–12% or 12% Bis-Tris, Thermo Fisher), transferred to Immobilon-P membranes, and blocked with 5% BSA or nonfat milk. Membranes were incubated with primary antibodies (anti-CDK10, 1:1000; anti-ETS2, 1:1000; anti-cPARP, 1:1000; anti-β-tubulin, 1:1000; anti-PolII pSer2, 1:1000; anti-PolII NTD, 1:1000 from Cell Signaling Technology, anti-PolII CTD (8WG16), 1:1000 from GENETEX, and anti-HSC70, 1:5000 from Santa Cruz) overnight at 4 °C, followed by HRP-conjugated secondary antibodies (1:10,000). Detection was performed using SuperSignal West Pico PLUS (Thermo Fisher) and imaged with ImageQuant 800 (Cytiva). ETS2 quantification was performed from 3 independent lysates using ImageQuantTL software (Cytiva).

### Fluorescence microscopy of GFP-tagged CDK10

OVCAR8 cells were transduced with lentivirus expressing GFP-tagged wildtype or kinase-dead CDK10, or control GFP. Three days post transduction, nuclei were live-stained by adding Hoechst 33324 into culture media at 1 µg/ml and cells were observed under the fluorescence microscope. Brightfield and fluorescence images were acquired using an epifluorescence microscope equipped with appropriate filter sets for Hoechst and GFP. Images were captured using identical exposure settings across conditions.

### Apoptosis assay by Annexin V staining

OVCAR8 and MDA-MB-231 cells were treated with CTX-439 and harvested at 24, 48, and 72 h post-treatment. Apoptosis was assessed using Annexin V staining (BioLegend, 640950) in 1× Annexin Binding Buffer (BioLegend, 422201) according to the manufacturer’s instructions with minor modifications. Briefly, cells were collected and washed twice with PBS, then resuspended at a density of 5 × 10⁶ cells/ml in 200 μL of 1× Annexin Binding Buffer containing Annexin V at a 1:200 dilution. Cells were incubated for 10 min at room temperature in the dark and subsequently analyzed by flow cytometry. The percentage of apoptotic cells was quantified based on Annexin V positivity.

### cDNA rescue assay

Cas9-expressing cells were first transduced with lentivirus expressing gCDK10-1 to knockout the endogenous CDK10, or gCTRL for intact CDK10. After puromycin selection, cells were further transduced with lentivirus produced with pKLV2-EF1a-neoT2AFlagCDK10wt-W, pKLV2-EF1a-neoT2AFlagCDK10m3-W, or a vector control pKLV2-EF1a-neoT2AFlagStop-W (EMPTY). Two to three days later, transduced cells were selected with G418.

### RNA-seq analysis

Total RNA was extracted using the RNeasy Mini Kit (Qiagen). RNA-seq libraries were prepared using the NEBNext Ultra II Directional RNA Library Prep Kit for Illumina (NEB), following the manufacturer’s protocol. The resulting libraries were sequenced on a DNBSEQ-G400RS platform using 100-base pair paired-end reads.

RNA-seq data from OVCAR8 and MDA-MB-231 cells (gCTRL or gCDK10-1) were mapped to hg38 using HISAT2 (v2.2.1). Gene quantification was performed using featureCounts (v2.0.3), and differential expression was analyzed with DESeq2 (v1.38.2). Genes with average read counts <1 were excluded. BigWig files were generated with deepTools (v3.5.1) and visualized in IGV. Efficacy evaluation values (EEVs) were calculating by dividing the TPM value of the last exon by that of the first exon of a given gene. Hierarchical clustering was performed based on log2 fold-change calculated by DESeq2 using Euclidean distance and Ward’s linkage. Genes were preselected based on the following criteria: LFC < −1, *p*adj < 0.01 between CTX-439 (90 nM for 6 h) and DMSO control in both CDK10-KO and control cells, and expressed at TPM > 1 in control cells. The RNA-seq dataset is deposited with Gene Expression Omnibus under the accession number GSE307213.

### RT-qPCR analysis

Total RNA was extracted using the RNeasy Mini Kit (Qiagen). 250 ng total RNA was reverse-transcribed using SuperScript IV First-Strand Synthesis System with an oligo-dT primer (Thermo Fisher). Quantification was performed using PowerUp SYBR Green Master Mix (Thermo Fisher) with the QuantStudio 3 apparatus (Applied Biosystems). The intron 12 signal was normalized to exon 12 using the ΔCt method. Primers are listed in Supplementary Table 5.

### ChIP-qPCR analysis

ChIP was performed on cells treated with DMSO or CTX-439 (90 nM, 6 or 24 h). Chromatin was crosslinked (1% formaldehyde), sonicated (Bioruptor II), and immunoprecipitated using anti-RNA Pol II Ser2P (Cell Signaling #13499) or IgG control. DNA was purified with the QIAquick PCR Purification Kit and quantified using SYBR Green qPCR (Thermo Fisher) on a QuantStudio 3 (Applied Biosystems). Primers are listed in Supplementary Table 5.

### Xenograft models

All animal experiments were conducted in an AAALAC-accredited facility and approved by the Institutional Animal Care and Use Committee (IACUC) of the Shonan Health Innovation Park. The study was performed in accordance with the Guide for the Care and Use of Laboratory Animals. A total of 1 × 10^7^ OVCAR8 gCTRL or gCDK10 cells were suspended in a 1:1 mixture of HBSS and Matrigel and injected subcutaneously into 6-week-old female SCID mice (CLEA Japan). Similarly, a total of 6 × 10^6^ MDA-MB-231 gCTRL or gCDK10 cells were injected subcutaneously into 6-week-old female BALB/cA nude mice (CLEA Japan). Tumors were measured every 3–4 days with calipers and volume calculated as: 0.5 × (length × width^2^). Once tumors reached ~100 mm³, mice were randomized into groups (*n* = 6/group) and treated with CTX-439 (7.5 or 15 mg/kg) or vehicle by oral gavage once daily for 10-21 days, during which, tumor volume was measured every 3–4 days. The CTX-439 formulation included DMSO, Cremophor EL, propylene glycol, and water (10:10:10:70, v/v/v/v). Tumor growth for each mouse was calculated as the change in tumor volume from baseline to the final measurement. Statistical analysis of tumor volume at the end of the experiment and tumor growth values was performed using one-sided Welch’s *t* tests with Holm correction. Adjusted *p*-values below 0.05 were considered statistically significant.

## Supplementary information

Supplementary Figures

Supplementary Tables

All the uncropped original western Blot data

## Data Availability

The RNA sequencing data generated in this study have been deposited in the Gene Expression Omnibus (GEO) under accession number GSE307213. Plasmids generated in this study have been deposited to Addgene and are available under accession numbers listed in the Methods section.
