# Supplementary material for: CDK10 loss primes tumor cells for transcriptional vulnerability and sensitizes to CDK12/13 inhibition
Source: Cell Death Dis. 2026 Jun 11;17(1):737. doi: 10.1038/s41419-026-08973-x (PMC13478345; doi:10.1038/s41419-026-08973-x)
Supplement: Supplementary file 1 [file 41419_2026_8973_MOESM1_ESM.docx]

**Supplementary Figure 1.** ***CDK10* KO validation and CTX-439 response assays in ES2 cells.** (A) Immunoblot analysis confirming *CDK10* KO in OV-90, OVCAR8, and ES2 cells transduced with gCDK10-1 or gCDK10-3. HSC70 was used as a loading control. (B) Baseline cell proliferation of *CDK10*-KO and control OVCAR8 cells, showing no significant difference. (C) Dose response assay in ES2 cells treated with CTX-439 for 72 h following *CDK10* KO (gCDK10-1, gCDK10-3). (D) Immunoblot analysis confirming re-expression of CDK10-wt or kinase-dead CDK10-m3 in *CDK10*-KO OVCAR8, OV-90, and ES2 cells following lentiviral transduction. (E) Dose response assay in ES2 cells reconstituted with empty vector, CDK10-wt, or kinase-dead CDK10-m3. Cells were treated with CTX-439 for 72 h. (F) Crystal violet staining of ES2 cells reconstituted with the indicated constructs and treated with CTX-439 for 4 days. Data are shown as mean ± SD (n = 3). ns, not significant by two-tailed Student’s t-test.

**Supplementary Figure 2. Validation of *CDK10* KO and cDNA rescue in MDA-MB-231 cells.** (A) Immunoblot analysis confirming CDK10 depletion in MDA-MB-231 and EVSA-T cells transduced with gCDK10-1. HSC70 was used as a loading control. (B) Baseline proliferation of *CDK10*-KO and control MDA-MB-231 cells. (C) Immunoblot analysis of CDK10 expression in *CDK10*-KO MDA-MB-231 cells transduced with empty, CDK10-wt, or kinase-dead CDK10-m3 vector. (D) Dose response assay in MDA-MB-231 cells reconstituted with empty, CDK10-wt, or kinase-dead CDK10-m3 vector and treated with CTX-439 for 72 h. (E) Crystal violet staining of MDA-MB-231 cells reconstituted with the indicated constructs and exposed to CTX-439 for 4 days. Data are shown as mean ± SD (n=3). Statistical significance was determined by two-tailed Student’s t-test. ns, not significant.

**Supplementary Figure 3. Validation of CDK10-loss mediated sensitization to CDK12/13 inhibition across breast cancer models.** (A) Immunoblot analysis of CDK10 protein levels in a panel of breast cancer cell lines. (B) Immunoblot confirmation of *CDK10* KO across a panel of seven breast cancer cell lines, including HCC1954, SUM149PT, MDA-MB-468, MDA-MB-436, MCF7, HCC1187, and T47D. HSC70 was used as a loading control. (C) Dose response assay in the same panel treated with CTX-439 for 72 h. (D) Dose response assay of SUM149PT treated with CTX-439 for 6 days. Data are shown as mean ± SD (n=3).

**Supplementary Figure 4. Transcriptomic alterations and validation of transcript truncation following *CDK10* KO and CTX-439 treatment.** (A) Subcellular localization of GFP-tagged CDK10-wt and kinase-dead CDK10-m3 in OVCAR8 cells by fluorescence microscopy. Nuclei were stained with Hoechst 33342. Scale bars, 50 μm. (B) Volcano plot of differential gene expression in OVCAR8 cells comparing *CDK10* KO (gCDK10-1) with control (gCTRL) under basal conditions. (C) Number of differentially expressed genes (DEGs) identified in OVCAR8 and OV-90 cells treated with CTX-439 at 10, 30, or 90 nM for 6 h. (D) RNA-seq track of *CAPN7* exon 12, intron 12, and exon 13, a magnified view of the relevant portion of Figure 5B. Target regions for qPCR were indicated. (E) Expression of *CAPN7* intron 12 relative to exon 12 was measured in control and *CDK10* KO cells treated with the indicated concentrations of CTX-439 for 6 h. Data are shown as mean ± SD (n=3). Statistical significance was analyzed using two-way ANOVA followed by Tukey’s multiple comparisons test. **p* < 0.05; ** *p* < 0.01, *** *p* <0.001; **** *p* <0.0001.

**Supplementary Figure 5. Enhanced elongation-associated truncation and prolonged transcriptional suppression in *CDK10* KO cells following CTX-439 treatment.** (A) Quantification of transcription elongation efficiency using efficiency evaluation value (EEV), defined as the ratio of transcription ending site (TES) depth to transcription starting site (TSS) depth. (B) Length-stratified analysis of EEV differences between *CDK10*-KO and control OVCAR8 cells treated with 30 nM CTX-439 for 6 h, showing reduced elongation efficiency in longer genes (>29 kb) in *CDK10* KO cells. *CAPN7* and *SENP6* genes are highlighted. Statistical significance was determined using the Wilcoxon paired test with Bonferroni correction for multiple comparisons. (C) Hierarchical clustering of 1,641 genes significantly downregulated at 6 h in both control and *CDK10* KO OVCAR8 cells treated with CTX-439 (log2FC < −1, padj < 0.01, and TPM > 1 in control cells), based on temporal log2 fold-change values (Euclidean distance, Ward’s linkage), identifying eight clusters. (D) Temporal expression trajectories of genes within each cluster shown in (C). Blue lines represent individual genes and orange lines indicate cluster mean log2 fold-change ± SD over time. Statistical significance was tested by paired t-test. (E) Gene body length distribution Statistical significance was determined by one-way ANOVA followed by Tukey’s multiple comparison test. **p* < 0.05; *** *p* <0.001; **** *p* <0.0001
