# Supplementary material for: CDK10 loss primes tumor cells for transcriptional vulnerability and sensitizes to CDK12/13 inhibition
Source: Cell Death Dis. 2026 Jun 11;17(1):737. doi: 10.1038/s41419-026-08973-x (PMC13478345; doi:10.1038/s41419-026-08973-x)
Supplement: Supplementary file 2 [file 41419_2026_8973_MOESM2_ESM.docx]

**Supplementary Table 1.** **Summary of top genetic hits from CRISPR-KO screen.** Table listing the top sensitizer genes from CRISPR-Cas9 screen across six cell lines. Data shown for genes identified in at least three models.

| **No. of cell line** | **Name of cell lines** | **No. of hit** | **Name of hit genes** |
| --- | --- | --- | --- |
| 6 | EVSAT MDAMB436 OV90 OVCAR8 SUM149PT T47D | 1 | CDK10 |
| 4 | EVSAT MDAMB436 OV90 OVCAR8 | 1 | CNOT4 |
| 4 | EVSAT OV90 OVCAR8 T47D | 1 | TSC2 |
| 4 | EVSAT OV90 OVCAR8 SUM149PT | 2 | LARP7 FAM58A |
| 4 | EVSAT MDAMB436 OV90 SUM149PT | 3 | TTC14 CDK12 CDKN2AIP |
| 4 | EVSAT OVCAR8 SUM149PT T47D | 1 | ADSS |
| 3 | EVSAT OV90 OVCAR8 | 2 | OTUB1 CUL5 |
| 3 | EVSAT MDAMB436 OV90 | 1 | HNRNPA2B1 |
| 3 | MDAMB436 OV90 SUM149PT | 1 | G6PD |
| 3 | EVSAT OV90 SUM149PT | 1 | GGNBP2 |
| 3 | EVSAT OVCAR8 SUM149PT | 2 | CMTR2 SCAF8 |
| 3 | EVSAT MDAMB436 SUM149PT | 1 | KHSRP |
| 3 | EVSAT SUM149PT T47D | 1 | RB1 |

**Supplementary Table 2. OVCA and BRCA cell lines used in this study**

| Ovarian cancer | Breast cancer |
| --- | --- |
| OV-90 | MDA-MB-436 |
| OVCAR8 | T47D |
| ES2 | EVSA-T |
|  | SUM149PT |
|  | HCC1954 |
|  | HCC1187 |
|  | MDA-MB-231 |
|  | MDA-MB-468 |

**Supplementary Table 3. gRNA list**

| Gene | gRNA ID | Sequence | Addgene ID |
| --- | --- | --- | --- |
| *CDK10* | V3_6-1 | TTCCATCACAGGGACCTGA | 246513 |
| *CDK10* | V3_6-3 | TGTGGGGAACCACCTGGAG | 246514 |
| *FAM58A* | V3_6-1 | GTCCCGGAGTTCCCAGAAG | 246515 |
| *FAM58A* | V3_6-5 | CTCCGCAGGTGTCAAGCTA | 246516 |
| *HNRNPA2B1* | V3_6-3 | GAATGAGGTCTTGCAGCCA | 246517 |
| *HNRNPA2B1* | V3_6-4 | TCACAGTTACATGAGCCCC | 246518 |
| *SCAF4* | V3_6-1 | GGCCTCCCTCAAGTGAAAC | 246519 |
| *SCAF4* | V3_6-6 | CGTTCTCGATCTCGGTCCA | 246520 |
| *TCC14* | V3_7-4 | GCTCTCTCCGATCCATACT | 246521 |
| *TCC14* | V3_7-5 | CTCTACAAGTGTCTGGCAG | 246522 |

**Supplementary Table 4. RT-qPCR primer list**

| Genes | Forward | Reverse |
| --- | --- | --- |
| *CAPN7_* Ex12 | GTCCTCATCACTGCGTCAAC | GCATATGCGTGTGTGGGAAC |
| *CAPN7*  Int12 | GGTGGGAAAGGAAATCATAGGG | ATTCCGCCTCAAAACTGCTG |

**Supplementary Table 5. ChIP qPCR primer list**

| Genes | Forward | Reverse |
| --- | --- | --- |
| *CAPN7_* TES+2kb | GAAGGGAGCCCTGGTCAAAA | TGTTTCCTGCCTCAATCCCC |
| *SENP6*_ TES+2kb | TAGTTCTGGTGCACACCTCC | TCACTTACCTTTGGGACCAGC |

**Supplementary Table 6. Antibodies used in this study**

| Antibodies | Supplier | Identifier |
| --- | --- | --- |
| CDK10 | Cell Signaling Technology | #36106 |
| ETS2 | Cell Signaling Technology | #66476 |
| HSC70 | Santa Cruz | #SC7298 |
| Cleaved PARP | Cell Signaling Technology | #9541 |
| β-Tubulin | Cell Signaling Technology | #2128 |
| Pol II pSer2 | Cell Signaling Technology | #13499 |
| Normal Rabbit IgG | MBL | #PM035 |
| POL II CTD (8WG16) | Genetex | #20817 |
| POL II NTD | Cell Signaling Technology | #14958 |
