# Supplementary material for: CDK10 loss primes tumor cells for transcriptional vulnerability and sensitizes to CDK12/13 inhibition
Source: Cell Death Dis. 2026 Jun 11;17(1):737. doi: 10.1038/s41419-026-08973-x (PMC13478345; doi:10.1038/s41419-026-08973-x)
Supplement: Supplementary file 3 [file 41419_2026_8973_MOESM3_ESM.docx]

**Original WB Data**
